# Chylomicrons produced by Caco‐2 cells contained ApoB‐48 with diameter of 80–200 nm

**DOI:** 10.14814/phy2.12018

**Published:** 2014-06-11

**Authors:** Andromeda M. Nauli, Yuxi Sun, Judy D. Whittimore, Seif Atyia, Guha Krishnaswamy, Surya M. Nauli

**Affiliations:** 1 Department of Pharmaceutical and Biomedical Sciences, College of Pharmacy, California Northstate University, Elk Grove, California; 2 Department of Health Sciences, College of Public Health, East Tennessee State University, Johnson City, Tennessee; 3 Department of Biomedical Sciences, James H. Quillen College of Medicine, East Tennessee State University, Johnson City, Tennessee; 4 Department of Internal Medicine, Division of Allergy and Clinical Immunology, James H. Quillen College of Medicine, East Tennessee State University, Johnson City, Tennessee; 5 Department of Pharmacology, College of Pharmacy, and Medicine, The University of Toledo, Toledo, Ohio

**Keywords:** Absorption, digestion, enterocytes, gastrointestinal, lipid

## Abstract

The small intestine generally transports dietary fats to circulation in triglyceride (TG)‐rich lipoproteins. The two main intestinal lipoproteins are chylomicron (CM) and very low‐density lipoprotein (VLDL). Unfortunately, studies on the CM biogenesis and intestinal transport of dietary fats have been hampered by the lack of an adequate in vitro model. In this study, we investigated the possible factors that might increase the efficiency of CM production by Caco‐2 cells. We utilized sequential NaCl gradient ultracentrifugation to isolate the CMs that were secreted by the Caco‐2 cells. To confirm the successful isolation of the CMs, we performed Fat Red 7B staining, TG reading, apolipoprotein B (ApoB) measurement, and transmission electron microcopy (TEM) analysis. We then tested the effects of cell differentiation, oleic acid, mono‐olein, egg lecithin, incubation time, and collagen matrix on CM secretion. We found that cell differentiation, oleic acid, and lecithin were critical for CM secretion. Using the Transwell system, we further confirmed that the CMs produced by our Caco‐2 cells contained significant amount of TGs and ApoB‐48 such that they could be detected without the use of isotope labeling. In conclusion, when fully differentiated Caco‐2 were challenged with oleic acid, lecithin, and sodium taurocholate, 21% of their total number of lipoproteins were CMs with the diameter of 80–200 nm.

## Abstract (precis)

When fully differentiated Caco‐2 cells were challenged with oleic acid, lecithin, and sodium taurocholate, they produced 21% chylomicrons that were 80–200 nm in diameter. These chylomicrons contained significant amount of ApoB‐48 and triglycerides such that they could be detected without the use of isotope labeling. Our studies identified factors that were important for chylomicron secretion.

## Introduction

Dietary fats, which mainly consist of triglycerides (TGs) and phospholipids (PLs), are digested in the lumen of the small intestine into monoglycerides (MGs), lysophospholipids (lysoPLs), and free fatty acids. In the presence of bile salts, these lipid digestion products form mixed micelles. Once taken up by the enterocytes, the MGs and lysoPLs will be re‐esterified to TGs and PLs, respectively; both will then be packaged into lipoproteins (Nauli and Nauli 2013). The two main intestinal lipoproteins are chylomicron (CM), commonly considered as lipoprotein with 80 nm or more in diameter, and very low‐density lipoprotein (VLDL), lipoprotein with less than 80 nm in diameter. Under normal physiological condition, CM particles can only be produced by the enterocytes (Nauli et al. 2006; Lo et al. 2008). This unique ability of enterocytes may be attributed to the high degree of ApoB lipidation and the high availability of lipids in the lumen of the digestive tract. Since the primary cells of the enterocytes have a short half‐life (Drover et al. 2005), it remains a challenge to come out with an adequate in vitro model.

Several studies have suggested that Caco‐2 cells can serve as an in vitro model to study CM biogenesis (Traber et al. 1987; Van Greevenbroek et al. 1996; Levy et al. 1999; Luchoomun and Hussain 1999). However, most of those studies rely on biochemical analysis with little to no microscopic evidence. Since lipoproteins tend to aggregate, the use of ultracentrifugation, gel filtration, or dynamic light scattering should ideally be complemented with microscopic analysis. The transmission electron microscopy (TEM) analysis with negative staining method is arguably a better method to obtain size distribution of the lipoproteins.

Our studies combined both biochemical and TEM analysis to investigate several factors that could improve the efficiency of CM secretion by Caco‐2 cells, namely cell differentiation, oleic acid (OA), mono‐olein (MO), egg lecithin, incubation time, and collagen matrix. We found that cell differentiation, OA, and egg lecithin could promote efficient CM production. We then compared our optimal condition with the previously reported condition that used less lipid and longer incubation time (Luchoomun and Hussain 1999). We found that our optimal condition led to a more efficient production of CMs. These CMs, which were around 80–200 nm in diameter, represented 21% of the total number of the isolated lipoproteins.

## Materials and Methods

### Caco‐2 cells

Caco‐2, human epithelial colorectal adenocarcinoma, cells (passage 17) were obtained from American Type Culture Collection (Manassas, VA) and were grown at 37°C with 5% CO_2_ in growth media (DMEM with 15% FBS). For propagation purposes, cells were split (1:6) when they have reached 50–70% confluence. Media was changed every other day. For the initial experiments, cells (passage 40–60) were grown in 10‐cm tissue culture dishes. For the later experiments, cells were grown on Tranwells (Cat. # 3420, 100‐mm dish, 3‐μm pore size, polycarbonate membrane; Corning, Inc., Tewksbury, MA). The prefiltered lipid mixture (unless specified, OA:lecithin:sodium taurocholate (NaTC) = 2:1.36:1.0 mmol/L) in 10‐mL growth media was added to the cells to induce the secretion of CMs. Cells that were grown in the tissue culture dishes were incubated with lipid mixture for 4 h, washed twice with PBS, and incubated with fresh growth media for 2 h to collect for the secreted lipoproteins. Cells grown on Transwells (Corning, Inc.) were incubated for 4 h with lipid mixture in the apical chamber and growth media without lipid mixture in the basolateral chamber. To isolate the CM and VLDL layers, the lipoprotein‐containing media were subjected to sequential NaCl density gradient ultracentrifugation (see Appendix). The successful isolation of these lipoproteins was confirmed by Fat Red 7B staining (Fig. A1), ApoB, TG, and TEM analysis (Fig. A2).

### TG measurement

The concentration of TGs was measured by using the colorimetric assay, as previously described (Nauli et al. 2003).

### Enzyme‐linked immunosorbent assay

High binding 96‐well plates (Cat. # 62409‐002; Thermo Scientific, Waltham, MA) were coated with monoclonal anti‐ApoB antibody (Cat. # HYB069‐02‐02; 100 μL in each well; 1:5000 dilution in the coating buffer [26 mmol/L Na_2_CO_3_, 23 mmol/L NaHCO_3_, pH = 9.2]; Pierce Antibody Products, Rockford, IL), washed with PBS‐0.5% Tween‐20 (PBS‐T), and then blocked with 5% BSA in PBS‐T. After washing, 100 μL of ApoB standard (Cat. # APOB25‐N‐100; Alpha Diagnostic International, San Antonio, TX) or sample was incubated in each well, washed, and added with the goat anti‐ApoB antibody (Cat. # 600‐101‐111; 1:1000; Rockland Immunochemicals Inc., Gilbertsville, PA). After washing, anti‐goat HRP‐conjugated secondary antibody (Cat. # 31402; 1:1000; Pierce Antibody Products) was incubated. The signals were detected by using the HRP substrate kit (Cat. # 172‐1064; Bio‐Rad, Hercules, CA) according to the manufacturer's suggested protocol.

### Transmission electron microscopy analysis

Samples were negatively stained by using freshly prepared 2% phosphotungstic acid, pH 6.0, as previously described (Nauli et al. 2006). They were examined by using Philips Technai 10 and representative pictures were taken.

### Experimental design

The experimental design is illustrated in Table 1. We tested the effects of cell differentiation, OA, MO, egg lecithin, incubation time, and collagen matrix on CM secretion. For collagen coating, each of the 10‐cm dish was incubated with 10 mL of collagen (Cat. # 160084; MP Biomedicals, Santa Ana, CA) dissolved in 20 mmol/L acetic acid; each dish was then washed gently with sterile water before being populated by Caco‐2 cells. The optimal condition (optimal [O]) obtained from the non‐Transwell experiments above was compared with the control group containing the same growth media but without lipid mixture (no lipid [NL]); and with the previously published condition (Luchoomun and Hussain 1999) containing less amount of lipid incubated over a longer period of time (less lipid [LL]). The comparison was performed in the Transwell system. Briefly, the growth media with (O and LL groups) or without lipid mixture (NL group) was added in the apical compartment; and the growth media without the lipid mixture was concurrently added in the basolateral compartment. At the end of the incubation period, the basolateral media was collected and subjected to NaCl density gradient ultracentrifugation.

**Table 1.**
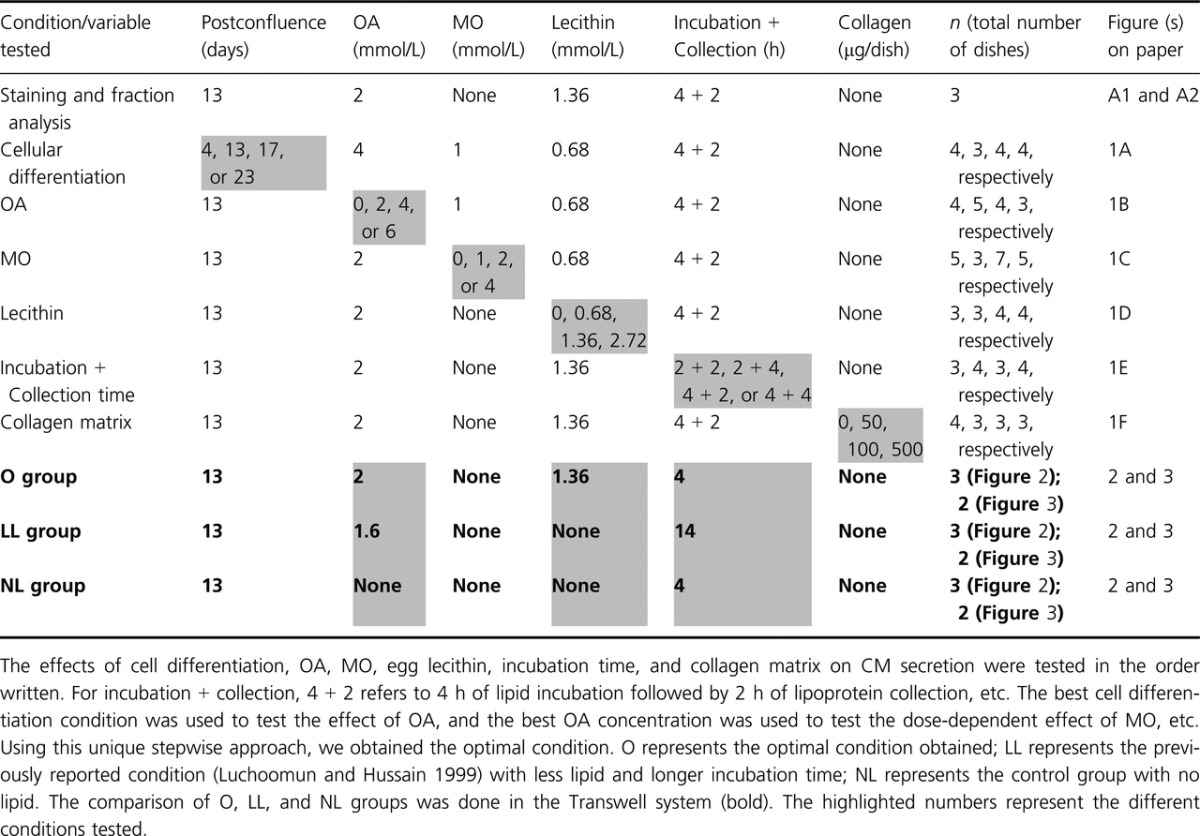
Experimental design

### Lipoprotein size analysis

For lipoprotein size and Western blot analysis, the basolateral media from O, LL, and NL groups were subjected to one‐step NaCl density gradient ultracentrifugation by skipping the first 30‐min spin at 10,000 rpm (see the Appendix). The combined CM and VLDL fraction was negatively stained (Nauli et al. 2006). The size of the lipoprotein particles was measured from the representative TEM images. At least 800 particles were counted.

### SDS polyacrylamide gel electrophoresis and immunoblotting

The lipoprotein fractions (10 μL) from O, LL, NL, and growth media control were isolated by one‐step NaCl density gradient ultracentrifugation. They were heated in reducing sample buffer and run on the 4–20% polyacrylamide gel (Cat# 456‐1096; Bio‐Rad). They were then transferred to nitrocellulose membrane and blocked with 5% skim milk in TBS‐T. The membrane was incubated with monoclonal anti‐ApoB antibodies (Cat. # HYB069‐02‐02; 1:5000 dilution in blocking buffer; Pierce Antibody Products), washed, and incubated again with anti‐mouse antibodies (Cat. # 31430; 1:5000; Pierce Antibody Products). The signals were detected by using the HRP substrate kit (Cat. # 34094; Pierce Antibody Products) according to the manufacturer's suggested protocol.

### Statistical analysis

The data shown are mean values ± standard errors (SE). To determine the statistical significance of three groups or more, one‐way ANOVA with multiple comparison tests was used; *t*‐test was used for comparison between two groups. Statistical analysis was performed in Excel (Microsoft, Redmond, WA) and was considered significant if *P *<**0.05. Unless specified, *n* ≥ 3 (*n* represents the total number of dishes; at least three separate experiments were performed).

## Results

### The effect of cell differentiation on CM secretion

**Figure 1.**
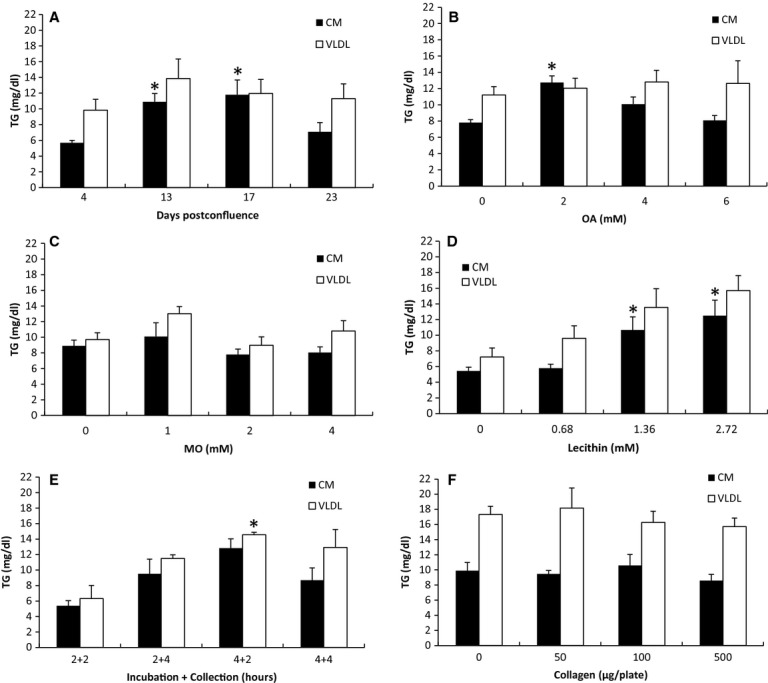
The effect of cell differentiation, OA, MO, lecithin, incubation time, and collagen matrix on lipoprotein secretion. (A) Cell differentiation: cells that were 4, 13, 17, or 23 days postconfluent were incubated for 4 h with 4 mmol/L OA, 1 mmol/L MO, 0.68 mmol/L lecithin, and 1 mmol/L NaTC in growth media (*n* = 4 for each; except for 13 days, *n* = 3). (B) OA: Thirteen‐day postconfluent cells were incubated for 4 h with 0, 2, 4, or 6 mmol/L OA in growth media containing 1 mmol/L MO, 0.68 mmol/L lecithin, and 1 mmol/L NaTC (*n* = 4 for 0 and 4 mmol/L; *n* = 5 for 2 mmol/L; *n* = 3 for 6 mmol/L). (C) MO: incubated with 0, 1, 2, or 4 mmol/L MO in growth media containing 2 mmol/L OA, 0.68 mmol/L lecithin, and 1 mmol/L NaTC (*n* = 5 for 0 and 4 mmol/L; *n* = 3 for 1 mmol/L; *n* = 7 for 2 mmol/L). (D) lecithin: incubated with 0, 0.68, 1.36, or 2.72 mmol/L lecithin in growth media containing 2 mmol/L OA and 1 mmol/L NaTC (*n* = 3 for 0 and 0.68 mmol/L; *n* = 4 for 1.36 and 2.72 mmol/L). (E) Time: incubated with 2 mmol/L OA, 1.36 mmol/L lecithin, and 1 mmol/L NaTC in growth media for either 2 or 4 h; and collected for either 2 or 4 h in growth media (*n* = 3 for 2 + 2 and 4 + 2; *n* = 4 for 2 + 4 and 4 + 4). (F) Collagen: Each 10 cm tissue culture dish was coated with 0, 50, 100 or 500 μg collagen before it was populated with cells (*n* = 3 for each; except 0 μg, *n* = 4). After the cells have reached 13‐day postconfluence, they were incubated for 4 h with 2 mmol/L OA, 1.36 mmol/L lecithin, and 1 mmol/L NaTC in growth media. The secreted lipoproteins were then collected for 2 h in growth media, and separated into CM and VLDL layers by sequential NaCl density gradient ultracentrifugation. The concentration of TG in the CM and VLDL layers was measured. Refer to Table 1 for the schematic representation of the experimental conditions. There were at least three separate experiments performed for each study. *n* represents the total number of dishes. All of the TG measurements were done in duplicates. Mean ± SE. One‐way ANOVA with multiple comparison tests was used (*P <**0.05 when compared with the groups without *. Comparisons were made only among the CM samples; among the VLDL samples; not between the CM and the VLDL samples).

Figure 1A shows that Caco‐2 cells that have reached 13 (10.90 mg/dL) and 17 days postconfluence (11.81 mg/dL) secreted CM more efficiently relative to those that have reached 4 (5.70 mg/dL) and 23 days postconfluence (7.09 mg/dL) (*P *=**0.0094). However, there was no statistical difference between 13 and 17 days postconfluence in the efficiency of CM secretion. The efficiency in VLDL secretion did not seem to be affected (9.84, 13.87, 11.95, and 11.32 for 4, 13, 17 and 23 days postconfluence, respectively). We have challenged Caco‐2 cells with lipid mixture prior to their confluence and discovered that the majority of them detached and died. When maintained under our specified condition, Caco‐2 cells generally did not live more than 24 days postconfluence.

### The effect of OA on CM secretion

Figure 1B shows that there was an effect of OA on CM secretion (*P *=**0.00017) with 2 mmol/L being the most effective concentration; no effect of OA on VLDL secretion was detected. When challenged with 0, 2, 4, and 6 mmol/L OA in lipid mixture, Caco‐2 secreted CMs with 7.81, 12.74, 10.08, and 8.08 mg/dL TG, respectively. The TG concentration in the VLDL layer for 0, 2, 4, and 6 mmol/L OA was 11.21, 12.04, 12.81, and 12.63 mg/dL, respectively. In our preliminary experiment, we have determined that 0.5 and 1 mmol/L OA resulted in lower efficiency of CM secretion compared to 2 mmol/L OA.

### The lack of dose‐dependent effect of MO on lipoprotein secretion

Figure 1C shows that there was no dose‐dependent effect of MO on CM and VLDL secretion. The TG concentration in the CM layer for 0, 1, 2, and 4 mmol/L MO was 8.91, 10.09, 7.79, and 8.06 mg/dL, respectively. The TG concentration in the VLDL layer for 0, 1, 2, and 4 mmol/L MO was 9.70, 13.01, 8.96, and 10.82 mg/dL, respectively.

### The dose‐dependent effect of lecithin on CM secretion

Figure 1D shows that there was a dose‐dependent effect of lecithin on CM secretion (*P *=**0.022) with 1.36 and 2.72 mmol/L being more effective than 0 and 0.68 mmol/L; there was no statistical difference between 1.36 and 2.72 mmol/L lecithin on CM secretion. Although there was no dose‐dependent effect of lecithin on VLDL secretion, the trend was clearly present (*P *=**0.053). The TG concentration in the CM layer for 0, 0.68, 1.36, 2.72 mmol/L lecithin was 5.42, 5.77, 10.65, and 12.48 mg/dL, respectively. The TG concentration in the VLDL layer for 0, 0.68, 1.36, and 2.72 mmol/L lecithin was 7.20, 9.59, 13.52, and 15.68 mg/dL, respectively.

### The effect of incubation time on VLDL secretion

Based on our previous studies in both mice and rats, it took the enterocytes 2–4 h to reach the maximum secretion of lipoproteins (Nauli et al. 2003, 2006). Therefore, in order to determine the most optimal incubation time, we subjected the cells with lipid mixture for either 2 or 4 h followed by an additional 2 or 4 h for lipoprotein collection. As shown in Figure 1E, the incubation and collection time was critical for VLDL secretion (*P *=**0.031) with 4 h of lipid incubation and 2 h of lipoprotein collection being the most effective; however, CM secretion did not seem to be significantly affected. The TG concentration in the CM layer for 2 h lipid incubation followed by 2 h lipoprotein collection (2 + 2), 2 + 4, 4 + 2, and 4 + 4 was 5.37, 9.50, 12.80, and 8.68 mg/dL, respectively. The TG concentration in the VLDL layer for 2 + 2, 2 + 4, 4 + 2, and 4 + 4 was 6.31, 11.48, 14.53, and 12.89 mg/dL, respectively.

### The lack of dose‐dependent effect of collagen coating on lipoprotein secretion

We tested the effect of collagen coating on lipoprotein secretion using the amount comparable with those from the previous reports (Schreider et al. 2002; Ratcliffe et al. 2009). We noticed that the rate of cell proliferation was consistently higher with as low as 50 μg collagen per dish. Therefore, we used the control group (0 μg collagen/dish) as the reference to determine the cell confluence. All of the four groups were simultaneously challenged with lipid mixture when the control group reached 13 days postconfluence (see Table 1). As shown in Fig. 1F, collagen coating did not affect lipoprotein secretion in a dose‐dependent manner. The TG concentration in the CM layer for 0, 50, 100, and 500 μg collagen/dish was 9.89, 9.47, 10.56, and 8.58 mg/dL, respectively. The TG concentration in the VLDL layer for 0, 50, 100, and 500 μg collagen/dish was 17.31, 18.17, 16.27, and 15.70 mg/dL, respectively.

### Higher efficiency of CM secretion relative to the previously reported condition

**Figure 2.**
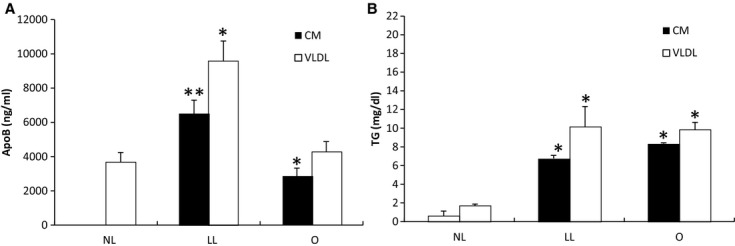
Comparing lipoprotein TG and ApoB generated under the optimal and the previously reported conditions. Cells were grown on Transwell until they reached 13‐day postconfluence. The apical compartment was added with growth media (NL group); 1.6 mmol/L OA and 0.5 mmol/L NaTC in growth media (LL group); or 2 mmol/L OA, 1.36 mmol/L lecithin, and 1 mmol/L NaTC in growth media (O group); and the basolateral compartment was concurrently added with the growth media. After incubating the cells for either 4 (O and NL groups) or 14 h (LL group), the basolateral media was subjected to sequential NaCl density gradient ultracentrifugation. The concentration of ApoB (A) and TG (B) was measured in both the CM and VLDL layers. There were three separate experiments conducted with one dish for each condition per experiment. All of the TG and ApoB measurements were done in duplicates. Mean ± SE. One‐way ANOVA with multiple comparison tests was used (*P <**0.05 when compared with the group without *; **P <**0.05 when compared with the group with and without *. Comparisons were made only among the CM samples; among the VLDL samples; not between the CM and the VLDL samples).

As shown by the experiments above, we have systematically obtained the optimal condition for efficient CM secretion. We applied the optimal condition to the Transwell system (Table 1; O group) so that it could be compared with the previously reported condition (Luchoomun and Hussain 1999), which used the Transwell system with less lipid and longer incubation time (Table 1; LL group). However, we had to alter their basolateral media from 0% or 0.1% FBS to 15% FBS because low serum resulted in very low TG and ApoB secretion and undetectable lipoproteins by TEM analysis (data not shown). In order to show that the lipoproteins that we detected (through TG, ApoB, and TEM analysis) were not from the serum (FBS) that was added to the media, we had a control group that was not challenged with lipid mixture but maintained the same amount of FBS in their media (Table 1; NL group). As shown by Fig. 2A, there were significant differences in the amount of ApoB in the CM layer (*P *=**0.004); each group was significantly different from the other two groups. The concentration of ApoB in the CM layer for NL, LL, and O groups was 0, 6510, and 2833 ng/mL, respectively. There were also significant differences in the amount of ApoB in the VLDL layers (*P *=**0.0007); however, only LL group was significantly different from the other two groups. The concentration of ApoB in the VLDL layer for NL, LL, and O groups was 3666, 9590, and 4282 ng/mL, respectively. Analysis of their TG revealed that both LL and O groups were significantly different from the NL group in both the CM (*P *=**0.0019) and the VLDL layer (*P *=**0.0014) (Fig. 2B). Assuming there was only one ApoB molecule per lipoprotein particle (Albers et al. 1996), our data suggest that the O group contained fewer lipoprotein particles but these lipoproteins were larger in diameter compared to those in the LL group; the NL group should only have small VLDL with little to no CM particles.

**Figure 3.**
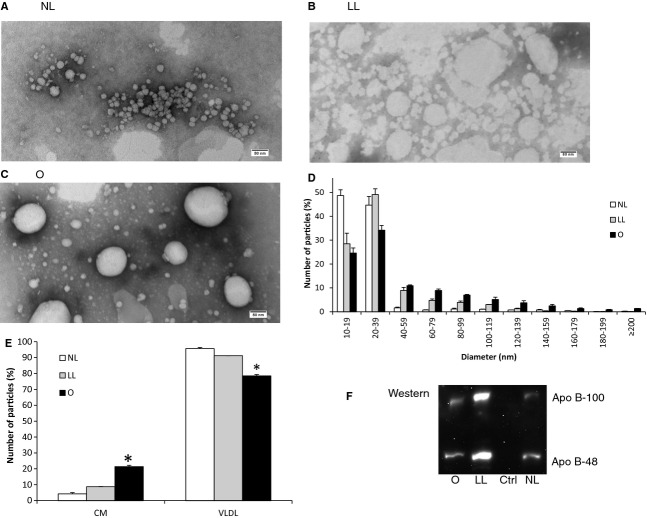
Comparing lipoprotein size and ApoB‐48 generated under the optimal and the previously reported conditions. Cells were grown on Transwell until they reached 13‐day postconfluence. The apical compartment was added with growth media (NL group); 1.6 mmol/L OA and 0.5 mmol/L NaTC in growth media (LL group); or 2 mmol/L OA, 1.36 mmol/L lecithin, and 1 mmol/L NaTC in growth media (O group); and the basolateral compartment was concurrently added with the growth media. After incubating the cells for either 4 (O and NL groups) or 14 h (LL group), the basolateral media was subjected to one‐step NaCl density gradient ultracentrifugation. The isolated lipoproteins from NL (A), LL (B), and O groups (C) were negatively stained, and their representative TEM images were depicted (scale bar = 80 nm). The size distribution of their lipoproteins was displayed in a histogram (D). The relative percentage of the number of CM particles (80 nm or larger) to the number of VLDL particles (smaller than 80 nm) was also depicted (E). Ten microliters of the lipoprotein fraction from each of the groups (from left: O, LL, growth media control, NL groups) was run on 4–20% polyacrylamide gradient gel under the reducing condition, transferred to nitrocellulose membrane, and blotted for ApoB. The top bands were ApoB‐100 and the bottom bands were ApoB‐48 (F). Two separate experiments were performed. Mean ± SE. One‐way ANOVA with multiple comparison tests was used (*P <**0.05).

Our biochemical data were, in fact, supported by our TEM analysis. NL group displayed small VLDL with insignificant number of CM particles (Fig. 3A); LL group displayed many VLDL and CM particles (Fig. 3B), but they were generally smaller than those from O group (Fig. 3C). Interestingly, the particles in the LL group were hazy probably due to a longer incubation time (14 instead of 4 h). To better discern the differences in their particle size, a histogram was generated (Fig. 3D). Based on the histogram, the relative percentages of the number of CM particles (80 nm or more in diameter) to the number of VLDL particles (smaller than 80 nm) were determined, as shown in Fig. 3E. The relative percentage of the number of CM particles to the number of the VLDL particles was significantly higher (*P *=**0.0006) in the O group (21.44%) than those in the NL (4.33%) and LL groups (8.80%); no significant difference was detected between NL and LL groups. To further compare the amount of ApoB‐48 relative to ApoB‐100, we performed Western blot analysis. As shown in Fig. 3F, there were slightly more ApoB‐48 than ApoB‐100 in all of the three groups. Importantly, ApoB could not be detected in the growth media control, ruling out the possibility that the ApoB measured was from the FBS/serum.

## Discussion

Caco‐2 cells undergo spontaneous differentiation and consequently display many biochemical and morphological characteristics of enterocytes (Chantret et al. 1988). Based on their ability to produce CMs, our studies suggest that Caco‐2 cells are fully differentiated once they have reached 13 days postconfluence. Importantly, the ability to produce CMs is relatively unique to Caco‐2 cells. To our knowledge, the other in vitro model capable of producing CMs is IPEC‐1 cell line overexpressing Apo A‐IV (Lu et al. 2006). Without the overexpression, IPEC‐1 cells produce insignificant amount of CMs when challenged with lipid. However, recent in vivo studies showed that Apo A‐IV KO mice secreted relatively more CMs than VLDLs (Kohan et al. 2012), indicating that Apo A‐IV might facilitate, but certainly not required for, CM formation.

Caco‐2 cells have been shown to produce CMs based on biochemical analysis (Traber et al. 1987; Van Greevenbroek et al. 1996; Levy et al. 1999; Luchoomun and Hussain 1999). As shown by our data (Fig. A2), even with the sedimentation coefficient of 4360 Svedberg units, a significant amount of large VLDLs (diameter <80 nm) was recovered. This could partly be due to the tendency of large VLDLs to aggregate (Nauli et al. 2006). Our data, therefore, suggest that the production of CMs by Caco‐2 cells might be overestimated if it was not analyzed by TEM.

CM formation is triggered by the presence of high amount of fatty acids in the luminal (apical) compartment of the enterocytes. Studies by Van Greevenbroek et al. (1996) showed that among fatty acids, OA was incorporated more efficiently into lipoproteins. Consistent with their studies, we showed that OA was essential for stimulating CM formation. However, exceedingly high concentration of OA (>6 mmol/L) was found to be detrimental to the cells. In contrast to OA, MO could not increase CM or VLDL secretion. Our data were in agreement with the previous studies that showed Caco‐2 cells did not primarily utilize MG in synthesizing TG for CM formation (Trotter and Storch 1993).

Similar to FA, lecithin was also a strong inducer of CM formation and had a tendency to induce VLDL secretion as well. Egg lecithin contains large quantity of PC (~80%) and phosphatidylethanolamine (~12%) with a small quantity of lysoPC and neutral lipids (Palacios and Wang 2005). Our TLC analysis confirmed the presence of PC and lysoPC in our egg lecithin (Fig. A3). Nakano et al. (2009) have shown that lysoPC, but not PC, stimulated VLDL secretion. Their studies showed negligible CM secretion probably because of the low OA concentration (0.6 mmol/L) used. However, our studies could not rule out the importance of other lipid components of egg lecithin in stimulating CM secretion.

Although the proliferation rate of Caco‐2 cells seemed to increase when they were grown on collagen‐coated matrix, their lipoprotein secretion was not affected. This could be explained by the fact that our Caco‐2 cells were already fully differentiated. Previous studies, however, showed that collagen matrix could increase ApoB secretion by Caco‐2 cells (Ratcliffe et al. 2009). Perhaps the increase in ApoB level observed in those studies was due to the difference in cell differentiation that was indirectly triggered by collagen coating.

The optimal condition obtained systematically from our studies resulted in the most efficient CM secretion by Caco‐2 cells, as supported by our ApoB, TG, and TEM analysis. Using our optimal condition, we showed that Caco‐2 could secrete CMs that were larger than 200 nm in diameter. The size of our CMs were comparable to those from the in vivo studies (Lo et al. 2008). However, Caco‐2 cells were only able to secrete up to 21% CM relative to the number of VLDL particles. Under the in vivo condition resembling normal postprandial state, the relative percentage of CM to VLDL could be up to 75% (Nauli et al. 2006). In contrast, under preprandial state the relative percentage of CM to VLDL could be as low as 5% (Lo et al. 2008). Therefore, Caco‐2 cells were capable of resembling postprandial CM secretion, albeit not reaching the maximum capacity observed in the in vivo studies.

There are several possible explanations as to why the relative percentage of the number of CM particles to the number of VLDL particles was lower in Caco‐2 cells than in the in vivo studies. First, Caco‐2 cells secrete both ApoB‐48 and ApoB‐100, as indicated by our Western blot analysis. Apobec‐1 KO mice, which produced only ApoB‐100, have been shown to secrete only about 30% CM relative to VLDL under the condition resembling normal postprandial state (Lo et al. 2008). The reduction in the relative percentage of the number of CM particles to the number of VLDL particles in Apobec‐1 KO mice strongly suggests that ApoB‐48 is more ideal than ApoB‐100 in facilitating lipid transport in the enterocytes. Second, Caco‐2 cells do not utilize MGs in synthesizing TGs (Trotter and Storch 1993), which could consequently lead to a lower availability of TGs to expand the core of CMs. Third, there could be some factors in the serum that are essential for CM formation. When Caco‐2 cells were supplemented with 0–0.1% FBS in the basolateral compartment over an extended period of time (14 h), lipoproteins could not be detected by TEM analysis; their ApoB and TG secretions were also very low. These factors, which are currently unknown, could be higher in the physiological serum than in the FBS. Fourth, the lipoproteins isolated in the in vivo studies were from the lymph and not from the lamina propria. Once released by the enterocytes into lamina propria, lipoproteins could enter either the blood or lymphatic vessels. It has been reported that the blood capillaries in the small intestine were permeable to particles as large as 30 nm in diameter (Simionescu et al. 1972). In essence, in vivo studies may not recover many of the small VLDLs that are recovered in the in vitro studies, as shown by their histograms of the particle size distribution (Nauli et al. 2006; Lo et al. 2008). In addition, as high as 39% of the infused TGs was estimated to enter the portal vein (Mansbach et al. 1991).

Caco‐2 cells may serve as a valuable in vitro model to study drug transport, particularly for the oral lipophilic drugs (Nauli and Nauli 2013). Due to their hydrophobicity, oral lipophilic drugs can be partitioned into CMs and VLDLs. As mentioned, small VLDLs may enter the blood capillaries in the lamina propria, subjecting them to liver metabolism before entering the systemic circulation. A better separation between CMs and VLDLs can be achieved by altering the ultracentrifugation conditions, which will allow the partitioning of drugs between CMs and VLDLs to be determined. This drug partitioning may be important to predict the bioavailability of oral lipophilic drugs.

In conclusion, we have shown that the fully differentiated Caco‐2 cells were capable of producing CMs of physiological size when they were challenged with lipid mixture consisting of FAs, lecithin, and bile salt. Although the relative percentage of the number of CM particles to the number of VLDL particles was lower compared to those in the in vivo studies, we believe that this was partly due to the partial recovery of small VLDLs in the in vivo studies. Our studies also indicated that biochemical analysis alone without the proper microscopic analysis might result in the overestimation of CM secretion.

## Acknowledgments

The authors would like to thank Roger Thompson, Jr., and Peter Laska for their preliminary work in determining the range of concentration and time to be tested in these experiments.

## Conflict of Interest

None declared.

## Figures and Tables

**Figure 4.**
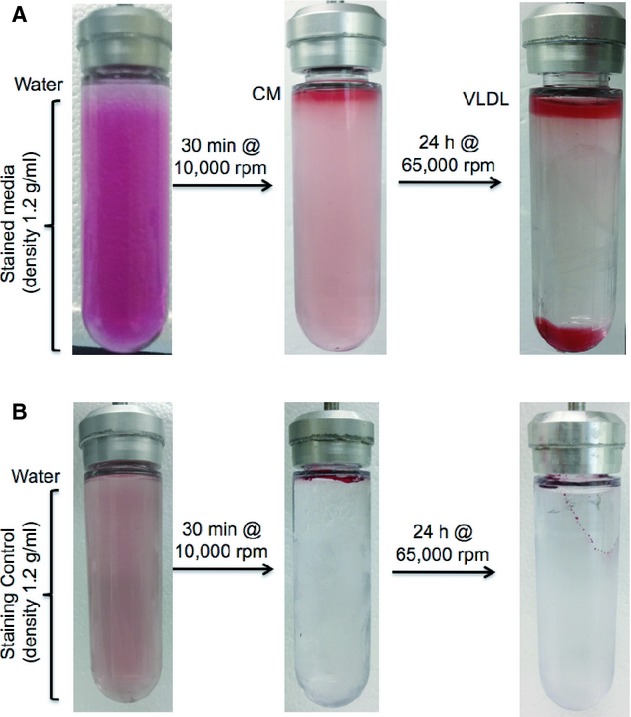
Fat Red 7B staining of the lipoprotein layers separated by sequential NaCl density gradient ultracentrifugation. (A) Thirteen‐day postconfluent cells were incubated for 4 h with 2 mmol/L OA, 1.36 mmol/L lecithin, and 1 mmol/L NaTC in growth media. The secreted lipoproteins were then collected for 2 h in colorless growth media and were stained with Fat Red 7B. The density of the prestained media was adjusted to 1.2 g/mL with NaCl and was carefully overlaid with 500 *μ*L of water. After a 30‐min spin at 10,000 rpm, the top ~500 *μ*L CM layer became heavily stained and the middle layer was lightly stained. The top 500‐*μ*L CM layer was immediately replaced with water and subjected to a 24‐h spin at 65,000 rpm. Notice that after the second spin, the top ~500 *μ*L VLDL layer and the bottom layer were heavily stained and the middle layer became clear. (B) Similar staining and spinning were performed, except with media without lipoproteins. The top low‐density layer was not stained at the end of each spin.

**Figure 5.**
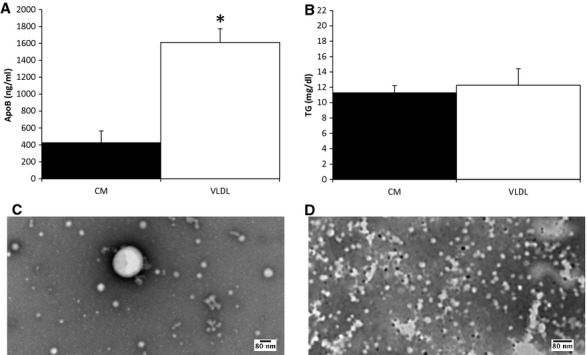
Analysis of the CM and VLDL layers by ELISA, TG assay, and TEM. Thirteen‐day postconfluent cells were incubated for 4 h with 2 mmol/L OA, 1.36 mmol/L lecithin, and 1 mmol/L NaTC in growth media. The secreted lipoproteins were then collected for 2 h in growth media and separated into CM and VLDL layers by sequential NaCl density gradient ultracentrifugation. The concentration of ApoB (A) and TG (B) in the CM and VLDL layers was measured. There were three separate experiments conducted (three dishes; *n* = 3). All of the TG and ApoB measurements were done in duplicates. Mean ± SE;* t*‐test was used (*P <**0.05). Representative TEM images of the lipoproteins in the CM (C) and VLDL (D) layers were also depicted. Scale bar = 80 nm.

**Figure 6.**
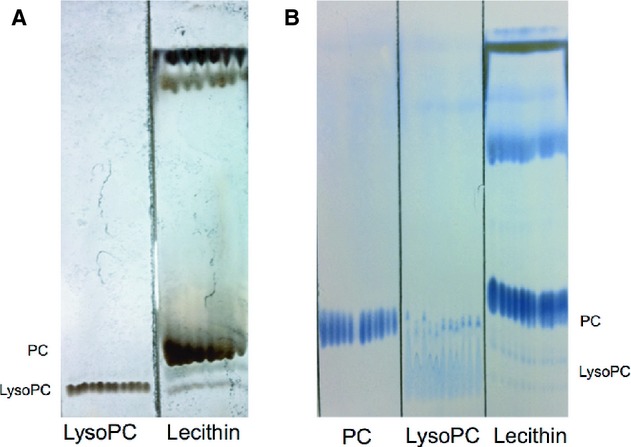
Determining the presence of lysoPC in egg lecithin by TLC. (A) 0.5 mg of lysoPC (left) and 2 mg egg lecithin (right) in chloroform were separated on silica gel 60 plates using chloroform:methanol:acetic acid:water (50:37.5:3.5:2) (v:v:v:v) as the solvent system. The plate was stained by choline staining followed by 20% sulfuric acid staining with 100°C heating until the color developed. (B) 0.5 mg PC (left), 5 mg lysoPC (middle), 0.5 mg lecithin (right) in chloroform:methanol:water (12:7:1) (v:v:v) were separated on silica gel 60 plates using chloroform:ethanol:water:triethylamine (30:35:7:3.5) (v:v:v:v) as the solvent system. The plate was stained by PL staining with 100°C heating until the color developed.

## Appendix

### Methods

#### Sequential NaCl density gradient ultracentrifugation

Media (basolateral media for Transwell) collected from cells were spun to remove cell debris, and then mixed with NaCl and water to obtain the density of 1.20 g/mL. The 1.20 g/mL density mixture was then carefully overlaid with 500 *µ*L of water and subjected to sequential ultracentrifugation. The top 500 *µ*L (CM fraction) was isolated by gentle pipetting immediately after the end of the first spin (30 min at 10,000 rpm; Thermo T865 rotor; equivalent to 4360 Svedberg units). The remaining mixture was similarly overlaid with 500 *µ*L of water and spun at 65,000 rpm for 24 hours (equivalent to 2.15 Svedberg units). The top 500 *µ*L of VLDL fraction was immediately isolated. It is important to note that the Svedberg units for CMs are >400. Larger CMs will have higher Svedberg units.

#### Fat Red 7B staining

For visualizing the lipoprotein layers on the ultracentrifugation samples, colorless media (without the pH indicator phenol red) was used to collect the secreted lipoproteins. The media was mixed with 200 *µ*L of Fat Red 7B solution (2 mg/mL Fat Red 7B in 0.1 mol/L NaOH with 5 *µ*L of Triton X‐100) and were subjected to sequential NaCl gradient ultracentrifugation as described above. Digital pictures were captured.

#### Thin layer chromatography analysis

The egg lecithin was analyzed by two different thin layer chromatography (TLC) methods to confirm the presence of lysophosphatidylcholine (lysoPC). In the first TLC, half milligram of lysoPC (Cat. # 100125‐446; left lane; Avanti Polar Lipids, Alabaster, AL) and 2 mg of egg lecithin (Cat. # IC10214625; right lane; MP Biomedicals, Santa Ana, CA) in chloroform were separated on silica gel 60 plates (Cat. # M57217; Fisher Scientific, Pittsburgh, PA) using chloroform:methanol:acetic acid:water (50:37.5:3.5:2) (v:v:v:v) as the solvent system. The plate was stained with choline staining (2 g potassium iodide, 4 mL glacial acetic acid, 0.34 g bismuth subnitrate in 100 mL total volume) followed by 20% sulfuric acid staining with 100°C heating until the color developed (from yellow to brown). In the second TLC, 0.5 mg PC (Cat. # P3556; left lane; Sigma‐Aldrich, St. Louis, MO), 5 mg lysoPC (middle lane), 0.5 mg egg lecithin (right lane) in chloroform:methanol:water (12:7:1) (v:v:v) were separated on silica gel 60 plates using chloroform:ethanol:water:triethylamine (30:35:7:3.5) (v:v:v:v) as the solvent system. The plate was stained by PL staining (1 g ammonium molybdate, 2 mL of 1 mol/L copper sulfate, 5 mL of concentrated sulfuric acid in 98 mL water) with 100°C heating until the color developed (blue). Digital pictures were captured for each TLC.

### Results

#### Detecting lipoproteins with Fat Red 7B

To visualize the lipoproteins before and after the NaCl gradient ultracentrifugation, we stained the colorless media collected from the Caco‐2 cells (see Table 1 for experimental condition) with Fat Red 7B. In our preliminary experiment, we have determined that Fat Red 7B stained TG, MG, and fatty acid, but not glycerol or bile salt (data not shown). As shown in Fig. A1 A, prior to the ultracentrifugation the lipoproteins were present throughout the media. The low‐density CM layer became visible at the end of the first spin. When the CM layer was replaced with water and subjected to the second spin, the VLDL layer became similarly visible. Interestingly, after the second spin the middle layer became clear suggesting that most of the lipoproteins have migrated away from the middle layer. The bottom layer was a gel‐like layer and upon examining under the TEM, there was no visible CM or VLDL. We have also performed Fat Red 7B staining on media without lipoproteins, and found out that the top 1.0 g/mL density layer was not stained at the end of the ultracentrifugation (Fig. A1 B).

#### Confirming the CM and VLDL layers by biochemical and TEM analysis

To further confirm the successful isolation of CMs and VLDLs, we analyzed their TG and ApoB content. As shown in Fig. A2 A, the amount of ApoB in the CM layer (425.30 ng/mL) was significantly lower (*P* = 0.005) than in the VLDL layer (1,612.18 ng/mL). The amount of TG, however, was comparable (11.27 mg/dL for CM; 12.27 mg/dL for VLDL) (Fig. A2 B). Since there was only one ApoB molecule per lipoprotein particle (Albers et al. 1996), our data suggested that the CM layer contained fewer lipoprotein particles, but these lipoproteins were larger in diameter compared to those in the VLDL layer. TEM analysis further confirmed that the CM layer (Fig. A2 C), in fact, contained larger lipoproteins relative to the VLDL layer (Fig. A2 D). Although CMs are commonly considered as lipoproteins of 80 nm or larger in diameter, ultracentrifugation with the sedimentation coefficient equivalent to 4360 Svedberg units (>400 are defined as CMs) resulted in the isolation of lipoproteins of <80 nm in diameter.

#### The presence of lysoPC in egg lecithin

Egg lecithin contains significant amount of PC, but its digestion product, lysoPC, is actually more important in stimulating lipoprotein secretion (Nakano et al. 2009). Therefore, we sought to determine whether lysoPC was present in our egg lecithin. In the first TLC analysis (Fig. A3 A), we separated the egg lecithin components and then stained for choline. Most of the choline was present in the PC fraction of the egg lecithin, but some was also present in the lysoPC fraction, as judged by the lysoPC standard. To further confirm its presence, we ran the second TLC and stained for PL. Figure A3 B showed that the majority of the PLs in the egg lecithin were in the form of PC, but some were also present in the form of lysoPC. Both of these TLC analyses indicate that lysoPC was present in the egg lecithin.

## References

[b1] Albers J. J. Kennedy H. Marcovina S. M. 1996 Evidence that Lp[a] contains one molecule of apo[a] and one molecule of apoB: evaluation of amino acid analysis data. [Online]. J. Lipid Res.; 37:192-196 http://www.ncbi.nlm.nih.gov/pubmed/8820114 8820114

[b2] Chantret I. Barbat A. Dussaulx E. Brattain M. G. Zweibaum A. 1988 Epithelial polarity, villin expression, and enterocytic differentiation of cultured human colon carcinoma cells: a survey of twenty cell lines. [Online]. Cancer Res.; 48:1936-1942 http://www.ncbi.nlm.nih.gov/pubmed/3349466 3349466

[b3] Drover V. A. Ajmal M. Nassir F. Davidson N. O. Nauli A. M. Sahoo D. 2005 CD36 deficiency impairs intestinal lipid secretion and clearance of chylomicrons from the blood. J. Clin. Invest.; 115:1290-1297. 15841205 10.1172/JCI21514 PMC1074677

[b4] Kohan A. B. Wang F. Li X. Bradshaw S. Yang Q. Caldwell J. L. 2012 Apolipoprotein A‐IV regulates chylomicron metabolism‐mechanism and function. Am. J. Physiol. Gastrointest. Liver Physiol.; 302:G628-G636. 22207575 10.1152/ajpgi.00225.2011 PMC3311309

[b5] Levy E. Yotov W. Seidman E. G. Garofalo C. Delvin E. Ménard D. 1999 Caco‐2 cells and human fetal colon: a comparative analysis of their lipid transport. [Online]. Biochim. Biophys. Acta; 1439:353-362 http://www.ncbi.nlm.nih.gov/pubmed/10446423 10446423 10.1016/s1388-1981(99)00085-2

[b6] Lo C.‐M. Nordskog B. K. Nauli A. M. Zheng S. Vonlehmden S. B. Yang Q. 2008 Why does the gut choose apolipoprotein B48 but not B100 for chylomicron formation? Am. J. Physiol. Gastrointest. Liver Physiol.; 294:G344-G352. 18006607 10.1152/ajpgi.00123.2007

[b7] Lu S. Yao Y. Cheng X. Mitchell S. Leng S. Meng S. 2006 Overexpression of apolipoprotein A‐IV enhances lipid secretion in IPEC‐1 cells by increasing chylomicron size. J. Biol. Chem.; 281:3473-3483. 16338933 10.1074/jbc.M502501200

[b8] Luchoomun J. Hussain M. M. 1999 Assembly and secretion of chylomicrons by differentiated Caco‐2 cells. Nascent triglycerides and preformed phospholipids are preferentially used for lipoprotein assembly. J. Biol. Chem.; 274:19565-19572. 10391890 10.1074/jbc.274.28.19565

[b9] Mansbach C. M. II Dowell R. F. Pritchett D. 1991 Portal transport of absorbed lipids in rats. [Online]. Am. J. Physiol.; 261:G530-G538 http://www.ncbi.nlm.nih.gov/pubmed/1887899 1887899 10.1152/ajpgi.1991.261.3.G530

[b10] Nakano T. Inoue I. Katayama S. Seo M. Takahashi S. Hokari S. 2009 Lysophosphatidylcholine for efficient intestinal lipid absorption and lipoprotein secretion in caco‐2 cells. J. Clin. Biochem. Nutr.; 45:227-234. 19794933 10.3164/jcbn.09-25 PMC2735637

[b11] Nauli A. M. Nauli S. M. 2013 Intestinal transport as a potential determinant of drug bioavailability. [Online]. Curr. Clin. Pharmacol.; 8:247-255 http://www.ncbi.nlm.nih.gov/pubmed/23343017 23343017 10.2174/1574884711308030012 PMC11957910

[b12] Nauli A. M. Zheng S. Yang Q. Li R. Jandacek R. Tso P. 2003 Intestinal alkaline phosphatase release is not associated with chylomicron formation. Am. J. Physiol. Gastrointest. Liver Physiol.; 284:G583-G587. 12466148 10.1152/ajpgi.00482.2002

[b13] Nauli A. M. Nassir F. Zheng S. Yang Q. Lo C.‐M. Vonlehmden S. B. 2006 CD36 is important for chylomicron formation and secretion and may mediate cholesterol uptake in the proximal intestine. Gastroenterology; 131:1197-1207. 17030189 10.1053/j.gastro.2006.08.012 PMC1994908

[b14] Palacios L. E. Wang T. 2005 Egg‐yolk lipid fractionation and lecithin characterization. J. Am. Oil Chem. Soc.; 82:571-578.

[b15] Ratcliffe D. R. Iqbal J. Hussain M. M. Cramer E. B. 2009 Fibrillar collagen type I stimulation of apolipoprotein B secretion in Caco‐2 cells is mediated by beta1 integrin. Biochim. Biophys. Acta; 1791:1144-1154. 19646550 10.1016/j.bbalip.2009.07.005 PMC4371871

[b16] Schreider C. Peignon G. Thenet S. Chambaz J. Pinçon‐Raymond M. 2002 Integrin‐mediated functional polarization of Caco‐2 cells through E‐cadherin–actin complexes. [Online]. J. Cell Sci.; 115:543-552 http://www.ncbi.nlm.nih.gov/pubmed/11861761 11861761 10.1242/jcs.115.3.543

[b17] Simionescu N. Simionescu M. Palade G. E. 1972 Permeability of intestinal capillaries. Pathway followed by dextrans and glycogens. [Online]. J. Cell Biol.; 53:365-392 http://www.pubmedcentral.nih.gov/articlerender.fcgi?artid=2108730&tool=pmcentrez&rendertype=abstract 4112540 10.1083/jcb.53.2.365 PMC2108730

[b18] Traber M. G. Kayden H. J. Rindler M. J. 1987 Polarized secretion of newly synthesized lipoproteins by the Caco‐2 human intestinal cell line. [Online]. J. Lipid Res.; 28:1350-1363 http://www.ncbi.nlm.nih.gov/pubmed/3430064 3430064

[b19] Trotter P. J. Storch J. 1993 Fatty acid esterification during differentiation of the human intestinal cell line Caco‐2. [Online]. J. Biol. Chem.; 268:10017-10023 http://www.ncbi.nlm.nih.gov/pubmed/8387510 8387510

[b20] Van Greevenbroek M. M. van Meer G. Erkelens D. W. de Bruin T. W. 1996 Effects of saturated, mono‐, and polyunsaturated fatty acids on the secretion of apo B containing lipoproteins by Caco‐2 cells. [Online]. Atherosclerosis; 121:139-150 http://www.ncbi.nlm.nih.gov/pubmed/8678919 8678919 10.1016/0021-9150(95)05712-9

